# Targeting Enox1 in tumor stroma increases the efficacy of fractionated radiotherapy

**DOI:** 10.18632/oncotarget.12845

**Published:** 2016-10-24

**Authors:** Clayton A. Smith, Stacey Mont, Geri Traver, Konjeti R. Sekhar, Peter A. Crooks, Michael L. Freeman

**Affiliations:** ^1^ Department of Radiation Oncology, Vanderbilt University Medical Center, Nashville, TN 37232, USA; ^2^ Current Address: Department of Radiation Oncology, Mitchell Cancer Institute, University of South Alabama, Mobile, AL 36604, USA; ^3^ Department of Pharmaceutical Sciences, College of Pharmacy, University of Arkansas for Medical Sciences, Little Rock, AR 72205, USA

**Keywords:** radiation therapy, Enox1, xenograft, tumor stroma, endothelial

## Abstract

The goal of this investigation was to clarify the question of whether targeting Enox1 in tumor stroma would synergistically enhance the survival of tumor-bearing mice treated with fractionated radiotherapy. Enox1, a NADH oxidase, is expressed in tumor vasculature and stroma. However, it is not expressed in many tumor types, including HT-29 colorectal carcinoma cells. Pharmacological inhibition of Enox1 in endothelial cells inhibited repair of DNA double strand breaks, as measured by γH2AX and 53BP1 foci formation, as well as neutral comet assays. For 4 consecutive days athymic mice bearing HT-29 hindlimb xenografts were injected with a small molecule inhibitor of Enox1 or solvent control. Tumors were then administered 2 Gy of x-rays. On day 5 tumors were administered a single ‘top-up’ fraction of 30 Gy, the purpose of which was to amplify intrinsic differences in the radiation fractionation regimen produced by Enox1 targeting. Pharmacological targeting of Enox1 resulted in 80% of the tumor-bearing mice surviving at 90 days compared to only 40% of tumor-bearing mice treated with solvent control. The increase in survival was not a consequence of reoxygenation, as measured by pimonidazole immunostaining. These results are interpreted to indicate that targeting of Enox1 in tumor stroma significantly enhances the effectiveness of 2 Gy fractionated radiotherapy and identifies Enox1 as a potential therapeutic target.

## INTRODUCTION

Radiation therapy, an important therapeutic modality for the treatment of cancer, is used to treat approximately 50% of cancer patients [1] for the purposes of local-regional control of invasive disease, to reduce the risk of metastases, and for palliation [2]. Current technology allows precise 3-dimensional irradiation of tumors that yields significant sparing of normal tissue. Yet even with this outstanding ability to selectively target tumor tissue, there are many instances in which tumors do not respond to radiation. Additionally, patients whose irradiated tumors recur following an initial response experience a significant reduction in median overall survival [3, 4]. The failure of radiation therapy to yield definitive, local-regional control in subsets of patients underscores the need for development of new therapeutic strategies.

Enox1 is a NADH oxidase [5, 6] that is expressed in endothelial and other cell types [6, 7]. Morpholino and pharmacological targeting of Enox1 in a zebrafish model of embryogenesis revealed that Enox1 is required for vascular development [6]. RNAi and small molecule targeting of Enox1 were shown to inhibit migration of human and mouse endothelial cells and the ability of these cells to form tubule-like structures in matrigel, as well as to suppress neo-angiogenesis driven by growth of Lewis Lung Carcinoma tumor cells in a dorsal skin fold vascular window chamber [8]. Additionally, pharmacological or RNAi-mediated targeting of Enox1 enhances endothelial cell susceptibility to the cytotoxic effects of ionizing radiation [8]. Thus, there is congruence between genetic approaches and small molecule inhibition with regard to inhibiting angiogenesis and the radiation response of endothelial cells. Multi-fractionation irradiation of allograft and xenograft tumors was found to synergize with pharmacological targeting of Enox1 to reduce tumor microvascular density and decrease tumor growth [8]. Thus, Enox1 enzymatic activity links NADH metabolism to both angiogenesis and the survival of endothelial cells following DNA damage produced by ionizing radiation.

The question of whether targeting of tumor stroma synergistically contributes to tumor control following fractionated radiotherapy represents a fundamental, unresolved question. The ‘top-up’ experimental approach was developed in order to amplify and detect intrinsic differences in radiation fractionation regimens [9]. Several small, clinically relevant doses of radiation are administered, which are then followed by a very large single dose that amplifies the effect of each fraction [9, 10]. Using this approach we investigated the question of whether concurrent x-irradiation and pharmacological inhibition of Enox1 in tumor stroma could increase the survival of HT29 tumor-bearing mice. The HT-29 tumor model was used because these cells do not express Enox1 and they are not radiosensitized by pharmacological inhibition of Enox1.

Herein, we report that pharmacological targeting of Enox1 inhibits DNA double strand break repair in endothelial cells and is one plausible mechanism for Enox1-mediated radiosensitization. To address the question of whether the survival of tumor-bearing mice could be increased by Enox1 targeting in tumor stroma during fractionated radiation therapy, HT-29 xenografts were administered 2 Gy x 4 q.d. in the absence or presence of an Enox1 small molecule inhibitor. On day 5 tumors were given a ‘top-up’ dose of 30 Gy. Targeting of Enox1 in tumor stroma increased survival of tumor-bearing mice 2 fold when measured 90 days after irradiation. This increase in survival, from 40% to 80%, was independent of the tumor's hypoxic fraction, as quantified by the hypoxic marker pimonidazole. We interpret these results to indicate that targeting Enox1 in tumor stroma concurrently with radiotherapy can provide a survival advantage.

## RESULTS

### Suppression of the NADH oxidase, Enox1

The activity of the NADH oxidase Enox1 is required for intracellular regulation of nicotinamide adenine dinucleotide homoeostasis. The enzyme catalyzes the following reaction: 2 NADH + O_2_ + 2H^+^ → 2 NAD^+^ + 2H_2_O [5]. Genetic or pharmacological inhibition of Enox1 has been shown to significantly increase intracellular NADH levels [6, 11]. Thus, it was of interest to determine if Enox1 inhibition would impact nicotinamide adenine dinucleotide-dependent metabolism.

**Figure 1:**
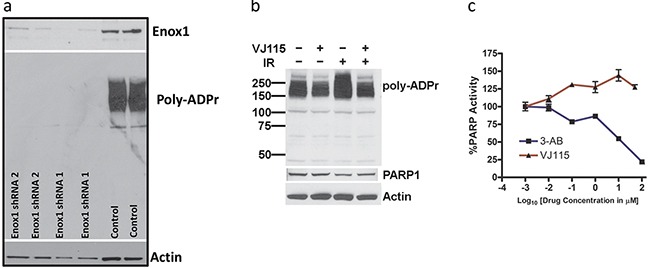
Targeting Enox1 inhibits PARP1 activity **a.** Poly(ADP-ribosyl)ation. HUVECs were transduced with retrovirus expressing scrambled, non-targeted shRNA or Enox1 shRNA. Poly(ADP-ribose) (Poly-ADPr) addition was measured by immunoblot using an antibody directed against Poly-ADPr. The immunoblot illustrates addition of Poly-ADPr primarily to PARP1. **b.** HUVECs were exposed to 50 μM VJ115 for 3 hrs, irradiated with (4 Gy), and then immediately processed for immunoblotting using an antibody directed against Poly-ADPr. The immunoblot illustrates addition of Poly-ADPr primarily to PARP1 **c.** VJ115 does not directly inhibit PARP1 activity. Purified recombinant PARP1 was exposed to varying concentrations of VJ115 or 3-AB. Enzyme activity was quantified using the Trevigen HT PARP assay kit according to the manufacturer's instructions.

Poly (ADP-Ribose) Polymerase 1, (PARP1) catalyzes poly(ADP-ribosyl)ation of proteins in a NAD^+^-dependent reaction and thus PARP1 activity is a useful surrogate marker for NAD+ metabolism. We used two independent Enox1 shRNAs expressed from retrovirus to suppress expression of Enox1 and then investigated PARP1 activity using a well characterized assay [12] (Figure 1a). Enox1 expression was completely suppressed 48 hrs after shRNA targeting, as was PARP1 activity. Cells respond to radiation-induced DNA strand breaks with rapid PARP1-dependent poly(ADP-ribosyl)ation. However, a 3 hr exposure to VJ115, ((Z)-(±)-2-(1-benzylindol-3-ylmethylene)-1-azabicyclo[2.2.2]octan-3-ol), a small molecule Enox1 inhibitor [8], suppressed radiation-induced PARP1 activity (Figure 1b). The basal poly(ADP-ribosyl)ation that was observed during the 3 hr VJ115 exposure may be a consequence of incomplete NAD+ depletion.

VJ115-mediated suppression of poly(ADP-ribosyl)ation was not a consequence of direct inhibition of PARP1 activity (Figure 1c). The activity of purified recombinant PARP1 measured in the presence of exogenous NAD+ was quantified in the absence or presence of the specific PARP1 inhibitor 3-aminobenzamide (3-AB) or VJ115. As shown in Figure 1c, 3-aminobenzamide produced concentration-dependent PARP1 inhibition relative to control activity (*P* < 0.05) whereas VJ115 did not produce inhibition (*P* > 0.05). Rather a small degree of enhanced activity (on the order of 25%) was observed. The reason is not currently understood. Taken together (Figure 1 and refs [6, 11]) the results indicate that targeting of Enox1 can deregulate nicotinamide adenine dinucleotide homeostasis.

### Enox1 and the radiation response

**Figure 2:**
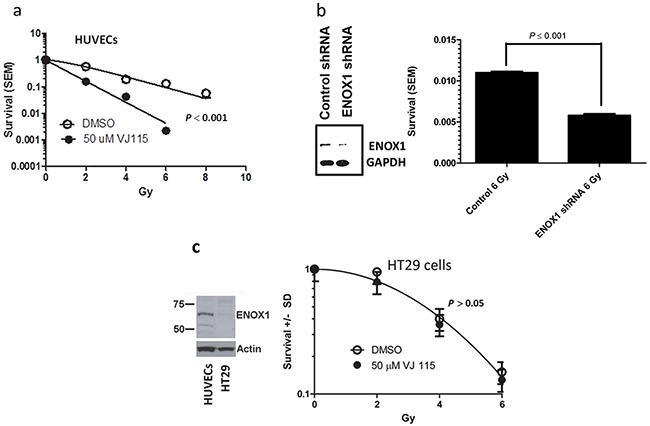
VJ115 radiosensitizes HUVECs **a.** Cell survival curves for HUVECs cultured overnight on 0.1% gelatin-coated dishes, exposed to 50 μM VJ115 or solvent control (DMSO/PBS) for 6 hrs, washed extensively and incubated for up to 3 weeks in colony formation assays. Cells were irradiated with ^137^Cs in the middle of the drug exposure. **b.** Cell survival of HUVECs transduced with retrovirus expressing scrambled, non-targeted shRNA or Enox1 shRNA. Forty-eight hrs later cells were either immunoblotted for Enox1 or irradiated with 6 Gy and subjected to a colony formation assay. **c.** HT-29 cells are not radiosensitized by VJ115. Immunoblot illustrating Enox1 expression in HUVECs but not in HT29 cells. HT29 cells were exposed to 50 μM VJ115 or solvent control (DMSO/PBS) for 6 hrs, washed extensively and incubated for up to 3 weeks in colony formation assays. Cells were irradiated with ^137^Cs in the middle of the drug exposure.

Although pharmacological and RNAi-mediated targeting of Enox1 increases endothelial cell radiosensitivity [8] as measured by colony formation assays, it is not known if this is a consequence of inhibition of the DNA damage response. Exposure to 1.5 Gy of x or γ-irradiation generates more than 1000 damaged bases, at least 1000 single strand DNA breaks, and 40 double strand breaks in a mammalian cell [13]. NAD+/NADH homeostasis represents a critical node for a cell's response to DNA damage [14]. Therefore we next determined whether targeting of Enox1 would affect repair of DNA damage. Human umbilical vein endothelial cells (HUVECs) were exposed to solvent control (DMSO) or 50 μM VJ115 for 3 hrs prior to, during, and for 3 hrs after irradiation. Previously, Enox1 was partially purified from HUVECs and using ENOX1 enzymatic activity assays we determined that the EC_50_ for VJ115 was 10 μM. We chose to use 50 μM in our cell culture assays in order to completely inhibit enzymatic activity. The resulting survival curves were best fit by the equation S = 1-(1-e^−D/Do)^)^n^ [15]. For the DMSO control survival curve, D_o_ = 2.1 and n = 1.7, (Figure 2a). Exposure to VJ115 decreased the D_o_ to 1.1 and n to 1.0, (*P* < 0.0001, extra sum of squares F test). Human microvascular endothelial cells (HMVECs) were also exposed to VJ115 and the resulting survival curves were fit to the equation S = 1-(1-e^−D/Do)^)^n^, Supplementary Figure S1. Inhibition of Enox1 in HMVECs did not affect the D_o_ but resulted in a statistically significant reduction in (n), from a value of 6.0 to 2.0 (*P* = 0.032, Supplementary Figure S1). Defective repair of DNA DSBs is reflected by decreases in D_o_ [16] and/or decreases in n [13]. Thus, in two endothelial cell lines pharmacological targeting of Enox1 produced statistically significant radiosensitization. To confirm that loss of Enox1 can radiosensitize endothelial cells, HUVECs were transduced with retrovirus expressing either scrambled, control shRNA or Enox1 shRNA. RNAi-mediated suppression of Enox1 was found to radiosensitize HUVECs (*P* < 0.001, Figure 2b). However, the radiation response of HT-29 cells, which do not express ENOX1 (Figure 2c), is independent of VJ115 (*P* > 0.05), suggesting that off target effects are minimal with regard to VJ115 -mediated radiation sensitization.

Although we have observed congruence between RNAi approaches and small molecule inhibition with regard to inhibiting angiogenesis and radiation sensitization, the remaining experiments focused on the small molecule inhibitor VJ115 in order to model preclinical xenograft studies where pharmacological targeting inhibits activity but does not cause loss of enzyme.

**Figure 3:**
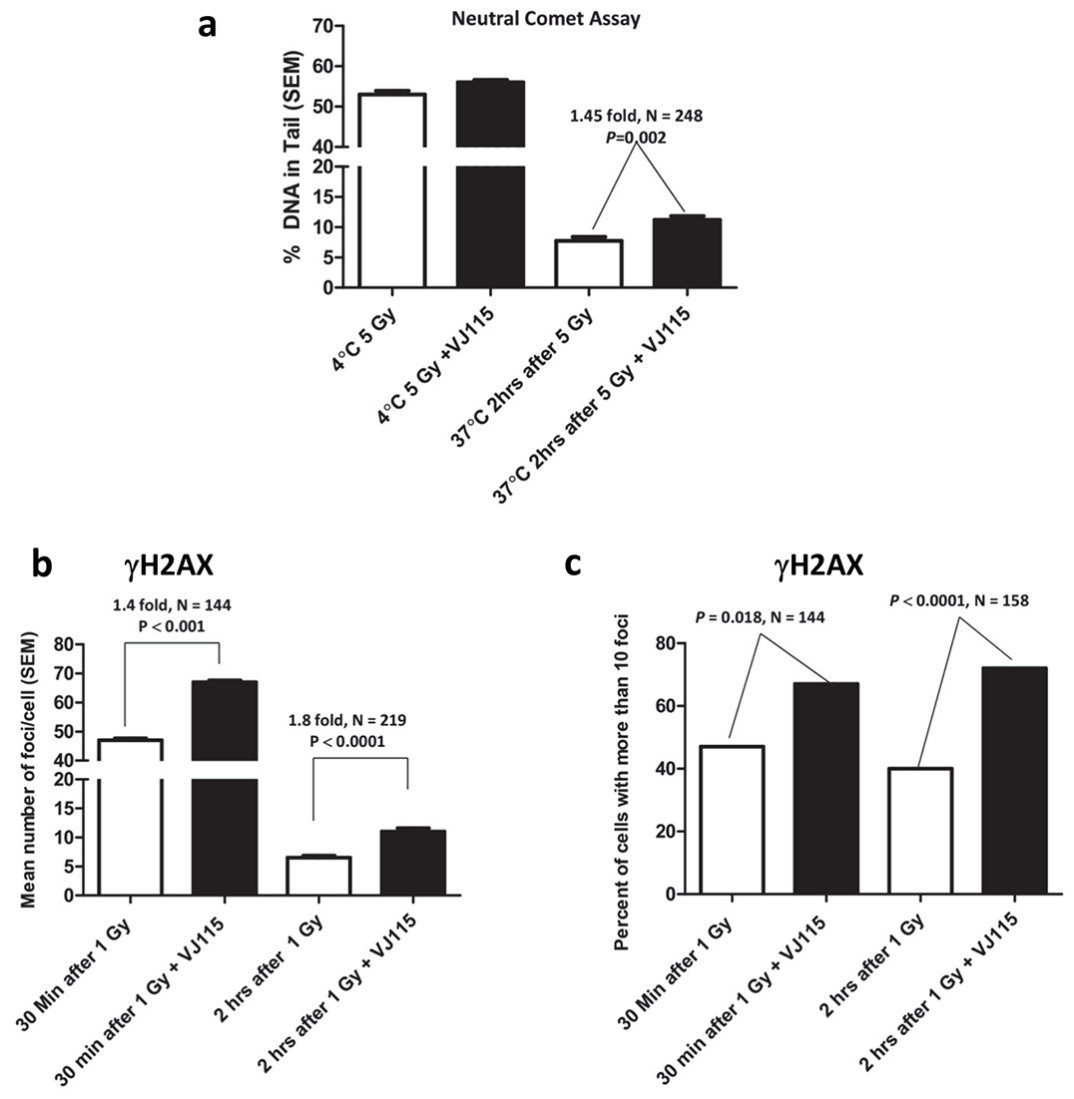
Inhibition of Enox1 impairs repair of DNA double strand breaks **a.** Neutral comet assay. HUVECs were exposed to 50 μM VJ115 for 1.5 hrs, placed on ice for 10 min, irradiated (5 Gy) on ice and then immediately processed for neutral comet assay. Alternatively cells were irradiated and then incubated at 37°C for 2 hrs prior to performing the neutral comet assay. **b** & **c.** γH2AX foci formation in HUVECs. Cells were exposed to 50 μM VJ115 at 37°C for 1.5 hrs, administered 1 Gy, incubated at 37°C for either 0.5 or 2 hrs, washed and fixed for immunofluorescence confocal microscopy. γH2AX foci per nuclei were quantified using ImageJ and are presented as mean number of foci per cell (b) or percent of cells with greater than 10 foci (c).

Impaired DNA double strand break repair represents a fundamental defect responsible for radiation-induced cell death [17]. Neutral comet assays are a well characterized and sensitive methodology for analyzing repair of DNA DSBs on a single cell basis [18]. Thus, neutral comet assays were employed to determine if Enox1-mediated radiosensitization was a consequence of repair defects. Analysis of comet tails (examples can be found in Supplementary Figure S2a) revealed that VJ115-mediated targeting of Enox1 in HUVECs did not affect the number of DNA DSBs generated during γ-irradiation performed at the non-permissive repair temperature of 4°C (Figure 3a). Repair was allowed to proceed after irradiation by incubating cells at the repair permissive temperature of 37°C. As shown in Figure 3a, repair of DNA DSBs was impaired in VJ115-treated cells. Two hours after irradiation we found that Enox1 targeting suppressed repair by 1.45 fold, *P* = 0.002.

γH2AX foci dynamics are a well-accepted surrogate for quantifying repair of DNA DSBs induced by ionizing radiation in single cells [19] (see Supplementary Figure S2b for examples). HUVECs were exposed to VJ115 for 1.5 hrs prior to and for 0.5 or 2 hrs after irradiation. Cells were fixed and nuclei imaged by immunofluorescence confocal microscopy. γH2AX foci were quantified on a per nucleus basis (Figure 3b). In addition the number of cells with more than 10 foci per nucleus was also quantified (Figure 3c). Non-irradiated cells contained 5 or fewer foci. Using both types of analysis we found that exposing HUVECs to pharmacological suppression of Enox1 resulted in statistically significant increases in the number of γH2AX foci per nucleus and the number of cells with more than 10 foci compared to solvent control (Figure 3b and 3c), consistent with results from the comet assay. Taken together we interpret these DNA damage assays to indicate that inhibition of Enox1 activity slowed the rate of DNA DSB repair, consistent with the increased radiosensitization observed in Figure 2.

**Figure 4:**
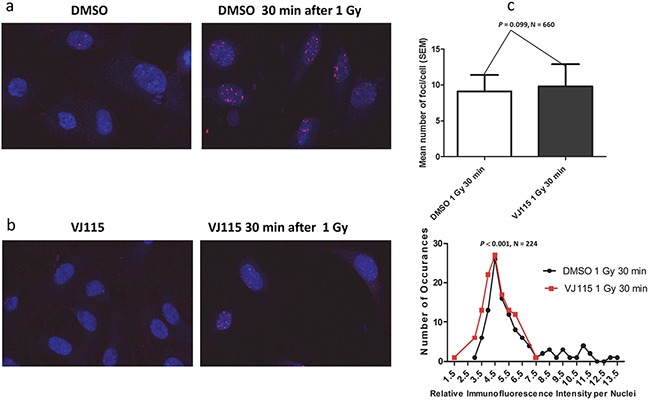
Exposure to VJ115 reduces 53BP1 accumulation at sites of DNA double strand breaks HUVECs were exposed to DMSO, solvent control **a.** or 50 μ M VJ115 **b.** for 1.5 hrs at 37°C, administered 0 or 1 Gy, incubated at 37°C for 0.5hrs, washed and fixed for immunofluorescence confocal microscopy. The number of 53BP1 foci per nuclei was quantified using ImageJ and is presented as mean number of foci per cell or as the relative immunofluorescence intensity per nucleus as a function of the number of occurrences (panel **c**). Relative immunofluorescence intensity per nucleus represents mean 53BP1 foci intensity per nucleus (N = 112 for DMSO control and N = 112 for VJ115).

During the repair process MDC1 is recruited to γH2AX foci, thus licensing recruitment of 53BP1 [20]. Interactions with H4K20me2 act to retain 53BP1 foci at sites of damage [21], where it participates in regulation of the DSB repair decision tree [22]. Impaired recruitment of 53BP1 to sites of DSBs can increase radiosensitivity [23]. We quantified the number of 53BP1 foci and relative foci immunofluorescence (IF) intensity per nucleus in HUVECs exposed to solvent control or the small molecule VJ115. Non-irradiated cells harbored 2 or less foci per nucleus, independent of VJ115 exposure (representative images shown in Figure 4a and 4b). Thirty min after administration of 1 Gy of γ-rays, cells contained an average of 9 ± 2 (SEM) 53BP1 foci per nucleus independent of Enox1 targeting (*P* = 0.099). However, a Mann Whitney analysis revealed that pharmacological inhibition of Enox1 activity was associated with a reduction in 53BP1 IF intensity per nucleus *(P* = 0.0128, N = 224, Figure 4c, lower panel). In order to better characterize this finding, the frequency of occurrence for each relative IF intensity value per nucleus was tabulated (Figure 4c, lower panel). The frequency distribution analysis indicated that there were 2 times more cells with IF values of 4.0 or less in irradiated cells exposed to VJ115 compared to irradiated cells exposed to solvent control (*P* = 0.023, Fisher’ exact test). We interpret these data to indicate that inhibiting Enox1 activity does not stop recruitment of 53BP1 to DNA DSB repair foci, but the amount of 53BP1 recruited to foci is significantly diminished.

### Targeting Enox1 in tumor stroma during fractionated irradiation

We have determined that HT-29 human colorectal adenocarcinoma cells are not radiosensitized by exposure to VJ115 (Figure 2c), whereas endothelial cells are (Figure 2b). This differential radiation response provided an opportunity to address a fundamental question: whether radiation sensitization of tumor stroma synergistically contributes to tumor control. Two experimental approaches were used to address this question.

**Figure 5:**
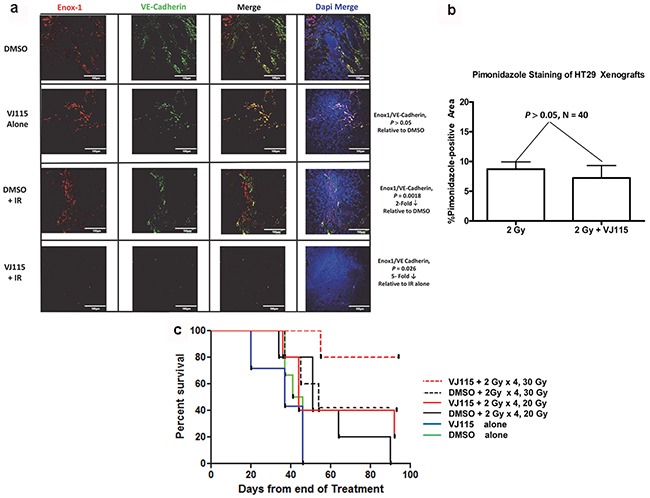
VJ115 radiosensitizes tumor vasculature and increases the survival of HT-29 tumor-bearing mice **a** and **b.** For 4 consecutive days mice bearing HT29 s.c. xenografts were injected i.p. with 40 mg/kg VJ115 or DMSO. Thirty min later tumors were administered 0 or 2 Gy of x-rays (300 kVp/10 mA, 1.55 Gy/min). On the 5^th^ day mice were injected i.p. with pimonidazole and then euthanized 60 min later. Tumors were excised, formalin fixed, paraffin embedded and immunostained with antibodies to Enox1 (a), VE-Cadherin (a), or Pimonidazole (b). Confocal microcopy was used to image Enox1 and VE-Cadhern at a magnification of 40x. White bars indicate 100μm distance. Pimonidazole whole slide imaging and quantification of immunostaining per tumor area were performed at a magnification of 20X by the Digital Histology Shared Resource at Vanderbilt University Medical Center. **c.** For 4 consecutive days mice bearing HT-29 s.c. xenografts were injected i.p. with 40 mg/kg VJ115 or DMSO. Thirty min later tumors were administered 0 or 2 Gy of X-rays (300 kVp/10 mA, 1.55 Gy/min). On day 5 tumors were administered a single fraction of 20 or 30 Gy. Survival was quantified for up to 90 days.

For 4 consecutive days HT-29 xenograft-bearing mice were injected i.p. with DMSO or 40 mg/kg VJ115. Thirty min after injection tumors (approximately 150 mm^3^) were administered either 0 or 2 Gy. On the 5th day mice were injected i.p. with 60 mg/kg pimonidazole and then euthanized 60 min later. Tumors were excised, formalin fixed, paraffin embedded, and immunostained with antibody to Enox1, VE-Cadherin or pimonidazole. Confocal microscopy was used to image Enox1 and VE-Cadherin immunostaining (Figure 5a), which is reported relative to DAPI staining (ie, on a per cell basis). We found that 4 daily injections of VJ115 did not affect expression of either Enox1 or VE-Cadherin compared to DMSO treatment (*P* > 0.05). Four daily 2 Gy fractions resulted in a statistically significant decrease in Enox1/VE-Cadherin immunostaining compared to DMSO treatment (≅2-fold, *P* = 0.0018). Four daily injections of VJ115 followed 30 min later by 2 Gy administered to the tumor resulted in a statistically significant decrease in Enox1/VE-Cadherin expression relative to radiation alone (≅5–fold, *P* = 0.026, Figure 5a). These data are interpreted to indicate that VJ115 significantly increased the effectiveness of the 2 Gy fractions.

Pimonidazole whole slide imaging (Supplementary Figure S3) and quantification of immunostaining per tumor area were performed at a magnification of 20X by the Digital Histology Shared Resource at Vanderbilt University Medical Center (www.mc.vanderbilt.edu/dhsr). An analysis indicated that the degree of tumor hypoxia was unaffected by administration of VJ115 alone. However, exposure to 4 daily 2 Gy fractions increased the degree of tumor hypoxia, consistent with the loss of tumor microvasculature observed in Figure 5a. Administration of VJ115 plus 2 Gy q.d. x 4 did not yield further increases in pimonidazole immunostaining (Figure 5b, *P* > 0.05, N = 6 fields per mouse, 5 mice per group). One interpretation for such a result is that VJ115-mediated radiosensitization occurred in areas that were already significantly hypoxic and the pimonidazole immunostaining assay was not sufficiently sensitive to detect further increases in hypoxia.

We next addressed the question of whether VJ115 targeting of Enox1 would affect tumor control. For 4 consecutive days HT-29 xenograft-bearing mice were injected i.p. with either DMSO or 40 mg/kg VJ115. Thirty min after injection tumors (approximately 150 mm^3^) were administered 2 Gy. On the 5^th^ day tumors were administered either a 20 or 30 Gy top-up dose in the absence of VJ115 (Figure 5c). The time between administration of VJ115 and the 2 Gy fraction was based on the pharmacokinetic profile of VJ115 (T_1/2_ in plasma = 30 min [24]).

Survival of tumor-bearing mice was quantified 90 days after the last treatment. No tumor-bearing mouse administered DMSO or VJ115 and sham irradiation remained alive 60 days after treatment (solid green or blue lines, Figure 5c). The short pharmacokinetic half-life of VJ115 may explain why 4 daily i.p. injections of VJ115 did not affect survival. Survival at 90 days was 20% or less for mice administered DMSO or VJ115, 2 Gy x 4 q.d., and a top-up dose of 20 Gy (solid black and red lines). However, 80% of tumor-bearing mice were alive at 90 days when they received VJ115 plus 2 Gy for 4 consecutive days followed by a top-up dose of 30 Gy (red dashed line, Figure 5c). The Bliss independence formula [E_IND_ = (E_A_ + E_B_) – (E_A_ x E_B_) and ΔE= E_OBS_ - E_IND_, where E_A_ is the effect of treatment A and E_B_ is the effect of treatment B] [25] was used to determine if this increase in survival was due to the two treatments being synergistic. The analysis indicated that ΔE was greater than 0 (*P* = 0.03, Fishers exact test). Thus the VJ115-mediated effect was considered synergistic with irradiation. We interpret these results to indicate that pharmacological targeting of Enox1 in tumor stroma inflicted a significant degree of radiation-induced damage to tumor stroma that contributed to radiation-induced tumor control.

## DISCUSSION

The NADH oxidase, Enox1, participates in NADH/NAD+ cell homeostasis [5]. Inhibition of Enox1 activity results in a profound elevation of intracellular NADH [6, 11], suppression of vascular development in zebrafish models of embryogenesis [6], inhibition of endothelial cell migration, abrogation of the ability to form tubule-like structures [8], and suppression of neoangiogenesis in dorsal skin fold vascular window chamber models [8]. Inhibiting Enox1 activity also enhances radiation-induced endothelial cell cytotoxicity, although the mechanism for this is undefined [8].

In the present study we demonstrate a direct link between Enox1 activity, repair of DNA damage, and radiation sensitivity. Inhibition of Enox1 activity diminished the repair of DNA DSBs, which translated into increased radiosensitization. When modeling cell survival curves using the equation S = 1-(1-e^−D/Do)^)^n^, the term n is a reflection of the ability to repair DNA damage [13]. Pharmacological targeting of Enox1 in both HUVECs and HMVECs produced a statistically significant decrease in n, which can be interpreted as a loss of DNA repair capacity. This concept was validated by quantifying γH2AX foci formation and carrying out neutral comet assays. While the mechanism responsible for loss of DNA repair capacity was not addressed in this study, we found that the amount of 53BP1 recruited to sites of DNA damage was significantly diminished. One possibility that could contribute to diminished 53PB1 accumulation and increased radiation sensitivity involves NAD+-PARP1-dependent generation of poly(ADP-ribose) (PAR) chains on histones. H4K20 methylation is required for recruitment of 53BP1 to sites of DNA DSBs [26]. H4K20 methylation requires mono ubiquitination of H4K91 by BBAP E3 and BAL1. PARP1 activity mediates BAL1-BBAP-mediated ubiquitinylation[27], which in turn regulates accumulation of 53BP1 to sites of DNA damage [28, 29]. Loss of PARP1 activity results in a failure to recruit BBAP to sites of damage, decreased 53BP1 accumulation, failure to resolve DNA DSBs, as measured by comet tail moment, and increased sensitivity to ionizing radiation [28, 29]. Testing of this hypothesis however, is beyond the scope of this investigation.

Previous studies have shown that small molecule inhibition of Enox1 in irradiated allograft and xenograft tumors significantly reduced tumor microvascular density and increased radiation-induced tumor growth delay [8]; however, it was not known if targeting Enox1 in stroma could extend the life of tumor-bearing mice. Therefore, we focused on the question of whether concurrent x-irradiation and pharmacological inhibition of Enox1 in tumor stroma could increase the survival of HT-29 tumor-bearing mice. The HT29 tumor model was used because these cells do not express Enox1 and they are not radiosensitized by pharmacological inhibition of Enox1. Thus, VJ115-mediated radiosensitization would be stroma-specific.

The majority of studies that have addressed the issue of whether targeting tumor stroma would increase tumor radiosensitivity have focused on angiogenic inhibitors and have used tumor growth delay as an endpoint [30–33]. While a combination of antiangiogenic treatment and irradiation produced significantly more growth delay than either modality by itself, it is hard to determine if the results are due to additivity or synergism. For example, Kozin et al [30] performed growth delay on two human xenografts using a VEGFR2 antibody. Because tumor grow delay studies do not quantify survival of tumor-bearing mice Tumor Control Dose (TCD) experiments were also undertaken. Tumor Control Dose (TCD)represents a dose of ionizing radiation needed to produce continuous control of irradiated tumors over a defined experimental interval, usually 90 or more days. Thus, a TCD_50_ value, for example, indicates that 50% of the tumor-bearing mice survived for a minimum of 90 days without tumor progression. Both the tumor growth delay experiments and the TCD analysis indicated that VEGFR2 antibody plus irradiation produced significant growth delay and decreased TCD values compared to either modality alone. However, Kozin and colleagues interpreted these results to be due to additive not synergistic effects, thus indicating that targeting of the VEGF/VEGFR pathway is not radiosensitizing.

Mice harboring targeted disruption of genes critical for repair of DNA damage also have been used to address the role of tumor stroma. Ogawa et al [34] used repair-proficient tumor cells implanted in wild type or SCID mice. Tumor growth delay induced by irradiation was significantly enhanced in SCID mice compared to tumor-bearing wild type mouse, suggesting that the stroma represents an important factor. Garcia-Barros and colleagues [35] used acid sphingomyelinase null and wild type mice to address the role of the tumor microenvironment. Endothelial cells in acid sphingomyelinase null mice are radiation resistant compared to sphingomyelinase proficient cells [35]. When wild type and acid sphingomyelinase null mice were implanted with repair proficient tumor cells radiation-induced tumor growth delay was found to be profoundly influenced by the endothelial radiation response [35]. However, alternative results were obtained from TCD experiments. Repair-proficient syngeneic and xenograft tumors were implanted into the hind legs of SCID or nude mice. Tumor-bearing legs were made totally anoxic/hypoxic by clamping the legs for 5 min and irradiating under clamped conditions. Quantification of TCD values led Budach et al [36] and Li et al [37] to conclude that the number of tumor progenitor cells and their intrinsic radiosensitivity were the major determinants of local tumor control, with little contribution from host stroma. However, a caveat to this interpretation is the potentially confounding effect of extreme hypoxia, a consequence of clamping, on DNA repair capacity. Extreme hypoxia has the potential to significantly impair the DNA damage response [38]. Thus, one cannot rule out an alternative interpretation that hypoxia incapacitated repair pathways in tumor stroma and negated the difference in DNA repair potential expected between SCID and nude mice.

In the experiments undertaken in this investigation we used the small molecule inhibitor VJ115 that targets Enox1 in the tumor vasculature but does not radiosensitize HT-29 tumor cells. We cannot, however, rule out radiosensitization of other components of the tumor microenvironment beyond the vasculature. We found that addition of the inhibitor to four daily 2 Gy factions of x-rays significantly decreased tumor vasculature and increased the survival of tumor-bearing mice compared to mice injected with DMSO control when the damage produced by the fractionated radiation was amplified by a 30 Gy top-up dose. It is important to note that we cannot rule out the possibility that 4 days of VJ115 administration by itself sensitized the tumor endothelial cells to the 30 Gy top-up dose and thus also contributed to the survival response.

Use of top-up doses is primarily a radiation biology laboratory method to quantitatively analyze the effects produced by low doses per fraction without the need to administer large numbers of fractions [9, 10]. We used this technique as a tool to address the central question of whether radiosensitization of tumor stroma would improve the ability of radiotherapy to cure tumors. While there is not a direct clinical correlate, a clinical analogy would be the use of a boost dose in patients with early stage breast cancer who undergo breast conserving therapy. Despite a high cure rate, invasive recurrence and death may occur in case of insufficient local treatment [39, 40]. Following breast conserving surgery, patients are administered a course of fractionated radiotherapy followed by a boost dose. The purpose of the boost dose is to reduce the risk of local relapse [40, 41]. Boost-induced normal tissue injury such as fibrosis represents a major limitation however. Ideally, one would like to administer a standard course of fractionated radiotherapy followed by a boost dose that controls local recurrence but does not promote normal tissue injury. We speculate that targeting tumor vasculature might provide significant local tumor control, allowing a boost dose to be decreased in order to spare local tissue from injury.

In summary, the data presented herein suggests that Enox1 regulation of tumor stroma radiosensitivity via NADH/NAD cellular metabolism can contribute significantly to tumor control and survival and identifies Enox1 as a potential therapeutic target.

## MATERIALS AND METHODS

### Antibodies

The following antibodies were used: anti-PAR was obtained from Tulip Biolabs catalog #1020; anti-γH2AX was obtained from Millipore catalog #05-636; anti-53BP1 was obtained from Novus Biologicals catalog #304; Anti-pimonidazole mouse IgG1 monoclonal antibody (MAb1) was obtained from Hypoxyprobe, Inc. Anti-Enox1 rabbit polyclonal antibody was custom made by Covance, Inc [11]; Anti-VE-Cadherin mouse monoclonal IgG1 antibody catalog #sc-9989 was obtained from Santa Cruz Biotechnology, Inc; Secondary antibodies Alexa 568 anti-rabbit goat polyclonal catalog #A11011 and Alexa 647 anti-mouse donkey polyclonal catalog #A31571 were both obtained from Life Technologies, Inc.

### Cell culture

HUVECs, and HT-29 cells were obtained from ATCC and were cultured according to ATCC directions. HMVECs were a gracious gift from DE Hallahan, Washington University School of Medicine. Colony formation assays were conducted as described previously [42].

### PARP1 activity

*In vitro* PARP1 activity Trevigen (Cat# 4676-096K) was measured using the manufacturer's directions.

### HT-29 xenografts

These studies were performed under the Guidelines for the Care and Use of Research Animals, Vanderbilt University Animal Studies Committee. Hind limbs of homozygous nu/nu athymic nude mice, approximately 6-8 weeks of age, were subcutaneously implanted with 2 × 10^6^ HT-29 human colorectal cancer cells. HT-29 tumors were allowed to reach a volume of approximately 150 mm^3^. For HT-29 tumors, 5 animals per group were used. Mice received daily i.p. injections of DMSO (25 μl) or 40 mg/kg of VJ115 in DMSO (25 μl) for 4 consecutive days, followed 30 min later by 0 or 2 Gy of x-rays (300 kVp/10 mA). During irradiation, mice were shielded such that only the tumors were irradiated. Tumors in the survival experiment were measured prior to initiation of treatment using digital calipers to ensure that there were comparable sizes between groups at the start. Tumors were deemed not cured if they exhibited any gross growth after 1 week following the top-up dose.

### Immunofluorescence staining and quantification of tumor sections

Tumors were excised, formalin-fixed and paraffin embedded. Tissue sections were cut at 5μm thickness and deparaffinized, permeabilized, and immunostained with Enox1 1:1,1000 (Alexa 568, red) and VE-Cadherin 1:200 (Alexa 647, green). For Enox1 and VE-Cadherin immunofluorescence confocal microscopy imaging was performed on 5 mice per group, 3 fields per mouse at a magnification of 40X at 0.66μM optical thickness, obtained using an Olympus FV-1000 inverted confocal microscope provided by the Cell Imaging Shared Resource at Vanderbilt University Medical Center. Quantification of immunofluorescence intensity was performed on ImageJ and corrected by DAPI staining on a per cell basis.

### Pimonidazole staining of tumor hypoxia

Pimonidazole was obtained from the Hypoxyprobe-1 Kit and used according the manufacturer's directions. Five mice per group and 6 fields per mouse were quantified following Pimonidazole immunohistochemistry.

## SUPPLEMENTARY MATERIALS FIGURES


