# Supplementary material for: Targeting Enox1 in tumor stroma increases the efficacy of fractionated radiotherapy
Source: Oncotarget. 2016 Oct 24;7(47):77926–36. doi: 10.18632/oncotarget.12845 (PMC5363632; doi:10.18632/oncotarget.12845)
Supplement: Supplementary file 1 [file oncotarget-07-77926-s001.pdf]

Targeting Enox1 in tumor stroma increases the efficacy of fractionated radiotherapy

SUPPLEMENTARY FIGURES

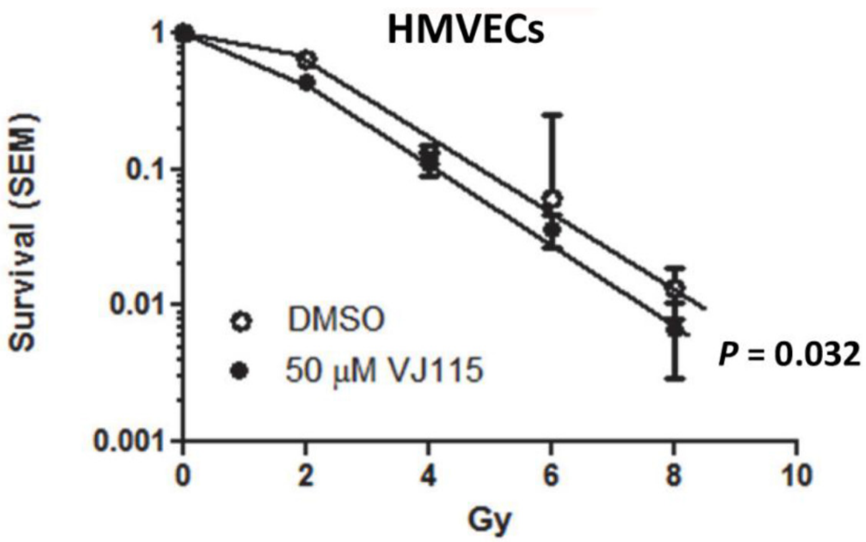

Supplementary Figure S1: Human microvascular endothelial cells (HMVECs) were exposed to 50 μM VJ115 3 hrs prior to, during, and for 3 hrs after irradiation. The resulting survival curves were fit to the equation $S = 1\text{-}(1\text{-}e^{-D/Do)})^{n}$.

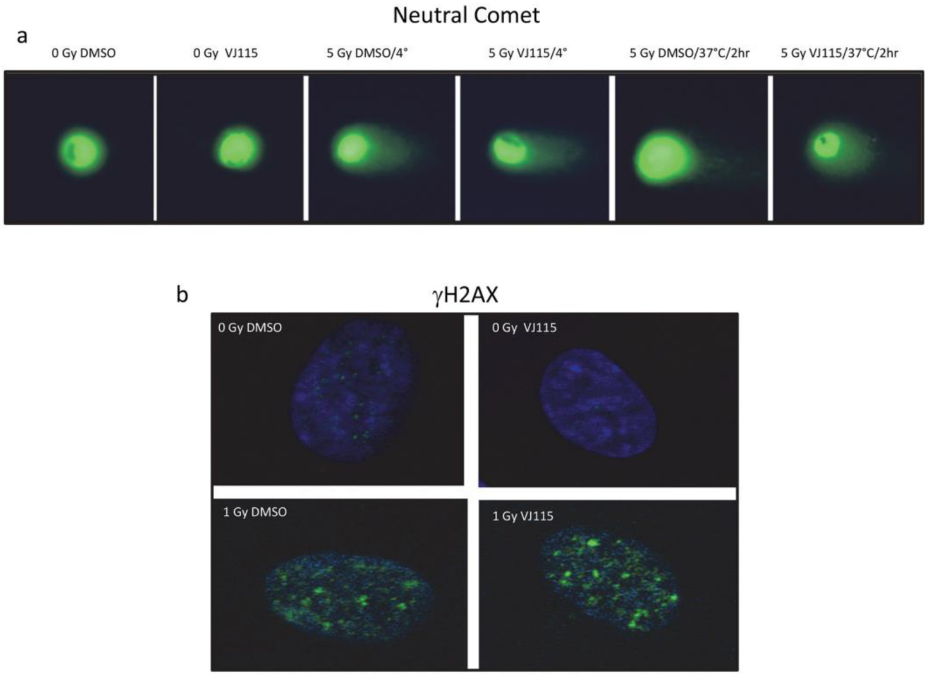

Supplementary Figure S2: Representative images of Neutral comet assays a. and γH2AX foci **b.** (a) HUVECs were exposed to 50 μM VJ115 for 1.5 hrs, placed on ice for 10 min, irradiated (5 Gy) on ice and then immediately processed for neutral comet assay. Alternatively cells were irradiated and then incubated at 37°C for 2 hrs prior to performing the neutral comet assay. b γH2AX foci formation in HUVECs. (b) Cells were exposed to 50 μM VJ115 at 37°C for 1.5 hrs, administered 0 or 1 Gy, incubated at 37°C for 0.5 hrs, washed and fixed for immunofluorescence confocal microscopy (63x). γH2AX foci (2° Ab green/FITC)/nuclei (DAPI).

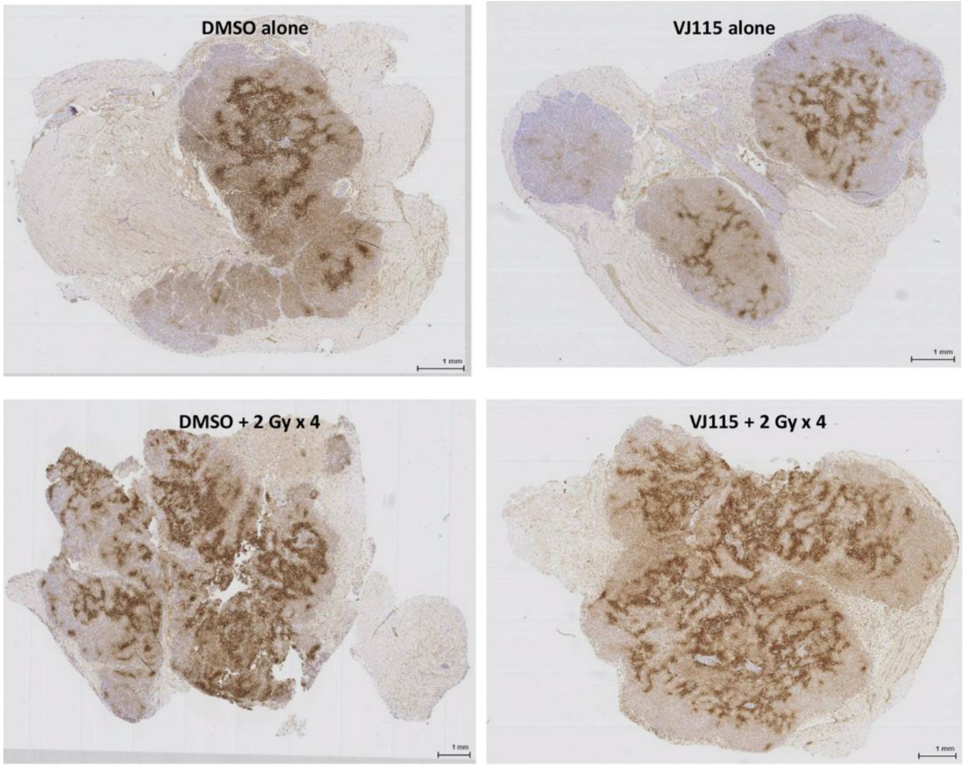

Supplementary Figure S3: IHC staining for the hypoxic marker pimonidazole. For 4 consecutive days HT29 xenograft-bearing mice were injected i.p. with DMSO or 40 mg/kg VJ115. Thirty min after injection tumors (approximately 150 mm^3) were administered either 0 or 2 Gy. On the 5th day mice were injected i.p. with 60 mg/kg pimonidazole and then euthanized 60 min later. Tumors were excised, formalin fixed, paraffin embedded, and immunostained with antibody to pimonidazole. Pimonidazole whole slide imaging and quantification of immunostaining per tumor area were performed at a magnification of 20X by the Digital Histology Shared Resource at Vanderbilt University Medical Center (www.mc.vanderbilt.edu/dhsr).
